# Supplementary material for: Emergence of transmissible SARS-CoV-2 variants with decreased sensitivity to antivirals in immunocompromised patients with persistent infections
Source: Nat Commun. 2024 Sep 18;15:7999. doi: 10.1038/s41467-024-51924-3 (PMC11411086; doi:10.1038/s41467-024-51924-3)
Supplement: Supplementary file 1 — Supplementary Information [file 41467_2024_51924_MOESM1_ESM.pdf]

Emergence of transmissible SARS-CoV-2 variants with decreased sensitivity to antivirals in immunocompromised patients with persistent infections

Supplementary Tables:

Supplementary Table 1. Antiviral treatment and samples sequenced.

Supplementary Table 2. Non-synonymous SARS-CoV-2 substitutions identified in nsp5 and nsp12.

Supplementary Table 3. Relevant laboratory data of patient 11595 during the disease course.

Supplementary Table 4. Immune and serum profiles of patient 16902 during the disease course

Supplementary Table 5. Immune and serum profiles of patient 17072 during the course of infection

Supplementary Table 6. Virus isolation from nasopharyngeal swabs of immunocompromised patients.

Supplementary Table 7. The IC_{50} values (μM) of nirmatrelvir and remdesivir against SARS-CoV-2 isolates WT and $nsp5^{T169I}nsp12^{V792I}$ in this study.

Supplementary Table 8. Binding energies (in kcal/mol) of nirmatrelvir drug molecule against nsp5 protein of WT and $nsp5^{T169I}nsp12^{V792I}$ isolates.

Supplementary Table 9. Binding energies (in kcal/mol) of remdesivir drug molecules against nsp12 protein of WT and $nsp5^{T169I}nsp12^{V792I}$ isolates.

Supplementary Table 10. Primers used for whole-genome sequencing of SARS-CoV-2 on the Oxford Nanopore technologies GridION platform.

Supplementary Figures:

Supplementary Figure 1. Longitudinal dynamics of non-synonymous SARS-CoV-2 variants.

Supplementary Figure 2. Isolation, growth kinetics and plaque size morphology of SARS-CoV-2 isolates.

Supplementary Figure 3. The SARS-CoV-2-$nsp5^{T169I}nsp12^{V792I}$ virus showed decreased sensitivity against nirmatrelvir and remdesivir therapies *in vitro*.

Supplementary Tables:

Supplementary Table 1. Antiviral treatment and samples sequenced.

Patient ID	Antiviral drug	Treatment day post-diagnosis	Days PCR +	Clade (PANGO)	Days pd	Ct	Accession
1995	RDV	1-10	28	20C (B.1.324)	0	NA	SAMN40750127
					1	NA	SAMN40750128
					6	NA	SAMN40750128
2646	RDV	15-19	32[+]	21K (BA.1)	0	NA	SAMN40750130
					26	25.6	SAMN40750131
10939	RDV	2-6, 14-18	33[‡]	20C (B.1.637)	13	16.5	SAMN40750132
					21	25.15	SAMN40750133
11595	RDV	2-7	38	20G (B.1.2)	14	19.4	SAMN40750134
					20	19.25	SAMN40750135
					26	20.7	SAMN40750136
					38	20.7	SAMN40750137
12105	RDV	16-20	58[‡]	20G (B.1.2)	0	14.8	SAMN40750138
					21	28.3	SAMN40750139
14675	Paxlovid	1-5	66	22C (BA.2.12.1)	0	32.3	SAMN40750140
	RDV	31-35			19	23.2	SAMN40750141
					24	22.5	SAMN40750142
					29	NA	SAMN40750143
					51	NA	SAMN40750144
16624	RDV	1-5	42	21K (BA.1)	0	NA	SAMN40750145
					12	NA	SAMN40750146
					27	22.7	SAMN40750147
16902	RDV	26-37, 47-51	50[‡]	21K (BA.1.15)	0	NA	SAMN40750148
					1	NA	SAMN40750149
					7	NA	SAMN40750150
					11	NA	SAMN40750151
					14	NA	SAMN40750152
					26	NA	SAMN40750153
					28	25.1	SAMN40750154
					36	NA	SAMN40750155
					42	NA	SAMN40750156
					47	23	SAMN40750157
16915	RDV	0-2	63	21K (BA.1.17.2)	0	NA	SAMN40750158
					14	20.83	SAMN40750159
					21	22.38	SAMN40750160
					35	19.8	SAMN40750161
17062	RDV	0-4	72	21K (BA.1.1)	8	NA	SAMN40750162
					11	18.76	SAMN40750163
17320	RDV	31-33	34	21K (BA.1.1)	30	22.4	SAMN40750170
					31	21.4	SAMN40750171
					34	25.5	SAMN40750172
17386	RDV	3-7, 38-42, 108-111, 148-152	157	21K (BA.1.1)	104	24.8	SAMN40750173
					141	NA	SAMN40750174
					143	20.8	SAMN40750175
17423	RDV	0-2	190	21K (BA.1)	0	NA	SAMN40750176
					3	21.5	SAMN40750177
					15	20.5	SAMN40750178
					32	14.5	SAMN40750179
					39	15.2	SAMN40750180
					42	28.2	SAMN40750181
					53	18	SAMN40750182
					60	23.4	SAMN40750183
					67	25.5	SAMN40750184
					81	19.7	SAMN40750185
					102	20	SAMN40750186
					137	20	SAMN40750187
					141	21.5	SAMN40750188
					152	NA	SAMN40750189
18323	RDV	74-78	131	21L	0	26	SAMN40750190

	Paxlovid	81-85		(BA.2)	6	17.1	SAMN40750191
					29	16.3	SAMN40750192
					73	NA	SAMN40750193
17072	Paxlovid	0-4	81	21K (BA.1.1)	22	NA	SAMN40750164
	RDV	20-24, 31-35, 63-67			52	21.9	SAMN40750165
					68	20.2	SAMN40750166
					77	NA	SAMN40750167
					80	25.7	SAMN40750168
					81	NA	SAMN40750169

Note: [+]indicates that the patient died in the following month after hospitalization without retesting for SARS-CoV-2. [‡]indicates that the patient died while SARS-CoV-2 positive. RDV-remdesivir. pd-post COVID-19 diagnosis. Ct-cycle threshold. NA-not available. Sequencing data are available under the BioProject PRJNA1088540.

Supplementary Table 2. Non-synonymous SARS-CoV-2 substitutions identified in nsp5 and nsp12. Samples are compared to their SARS-CoV-2 clade reference. Mutations exceeding 98% frequency of the sequence reads in all samples sequenced per patient may signify strain-specific diversity that established the infection, rather than fixation over the course of the infection.

Patient ID	Antiviral drug	Treatment day post-diagnosis	Clade (PANGO)	Total Collections Sequenced	Variant	Frequency Range	Number of Samples/Collections with Variant
1995	RDV	1-10	20C (B.1.324)	3	nsp12: A16V	1	3
10939	RDV	2-6, 14-18	20C (B.1.637)	2	nsp5: P96S	1	2
11595	RDV	2-7	20G (B.1.2)	4	nsp12: E136A	0.27	1
					nsp12: V166L	0.11	1
					nsp12: Q444K	0.05 - 0.07	2
					nsp12: V792I	0.09 - 0.31	3
					nsp12: M794I	0.17 - 0.9	4
					nsp12: C799F	0.1	1
					nsp12: V820G	0.03	1
12105	RDV	16-20	20G (B.1.2)	2	nsp12: V605I	1	2
16902	RDV	26-37, 47-51	21K (BA.1.15)	10	nsp12: C464Y	1	2
					nsp12: C799Y	0.98	1
16915	RDV	0-2	21K (BA.1.17.2)	4	nsp12: T644M	0.06 - 0.07	2
17072	Paxlovid	0-4	21K (BA.1.1)	6	nsp5: M165I	0.09	1
	RDV	20-24, 31-35, 63-67			nsp5: T169I	0.34 - 0.92	4
					nsp5: A173T	0.1 - 0.74	2
					nsp5: G283C	0.05	1
					nsp12: T643I	0.26	1
					nsp12: V792I	0.27 - 0.98	4
					nsp12: E796K	0.23 - 1	3
17320	RDV	31-33	21K (BA.1.1)	3	nsp12: I171M	0.05	1
17423	RDV	0-2	21K (BA.1)	14	nsp5: L253I	0.03 - 0.04	2
					nsp12: V820G	0.03	1
					nsp12: F859L	0.03	1

18323	RDV	74-78	21L (BA.2)	4	nsp5: T21I	0.04	1
	Paxlovid	81-85*					

** Antiviral treatment course occurred after all samples were collected and sequenced.*

Supplementary Table 3. Relevant laboratory data of patient 11595 during the disease course.

Laboratory Parameter	Days after COVID-19 Diagnosis								
	Normal Range	0	7	14	20	23	26	30	38
CT value				19.4	19.25		20.7		20.7
WBC count, ($\times 10^3$/uL)	3.4-11.2	3.79	2.33	2.8	1.47	1.45	1.62	3.98	4.02
Neutrophil count, ($\times 10^3$/uL)	1.8-7.0	**	**	2.38	1.22	0.85	1.38	3.0	3.59
Neutrophils (%)	45-75	**	**	85	83.1	82	81	93	89.4
Lymphocyte count, ($\times 10^3$/uL)	1.18-3.74	**	**	0.22	0.13	0.08	0.16	0.08	0.08
Lymphocytes (%)	20-50	**	**	8	8.8	8	10	2	2
D- Dimer (ng/mL)	0-229	**	**	**	**	451	**	**	**
CRP (mg/dL)	<=0.9	**	**	**	**	19.0	**	**	**
ESR (mm/hr)	0-20	**	5	**	**	25	**	**	**
LDH (U/L)	118-230	**	321	**	280	375	317	444	**

Abbreviations. CRP: C-reactive protein; CT: cycle threshold; ESR: Erythrocyte sedimentation rate; LDH: Lactate dehydrogenase; WBC: White blood cells; ** Indicates that these laboratory values were not available.

Supplementary Table 4. Immune and serum profiles of patient 16902 during the disease course.

Laboratory Parameter	Normal Range	Days after COVID-19 Diagnosis								
		**0**	**1**	**7**	**11**	**14**	**28**	**36**	**47**	**52**
CT value							25.1		23	
WBC count, ($\times10^3$/uL)	3.4-11.2	1.0	1.22	5.8	2.2	2.8	3.53	4.17	1.03	1.63
Neutrophil count, ($\times10^3$/uL)	1.56-6.13	0.6	0.83	4.2	1.5	2.1	2.93	3.59	0.83	1.55
Neutrophils (%)	45-75	61.4	66	80	66	80	83	86	79	95
Lymphocyte count, ($\times10^3$/uL)	1.18-3.74	0.2	0.17	0.52	0.37	0.28	0.19	0.17	0.08	0.08
Lymphocytes (%)	20-50	18.5	14	9	16	10	5.4	4	8	6
D- Dimer (ng/mL)	0-229	**	**	**	**	**	160	338	175	1588
CRP (mg/dL)	<=0.9	**	9.2	**	**	**	7.9	0.7	0.4	0.4
LDH (U/L)	118-230	273	274	523	312	325	414	363	502	575
Procalcitonin (ng/mL)	<=0.08	**	**	**	**	**	**	**	**	**

Abbreviations. CRP: C-reactive protein; CT: cycle threshold; ESR: Erythrocyte sedimentation rate; LDH: Lactate dehydrogenase; WBC: White blood cells; ** Indicates that these laboratory values were not available.

Supplementary Table 5. Immune and serum profiles of patient 17072 during the course of infection

Laboratory Parameter	Normal Range	**Days post-diagnosis**																
		-2	22	23	24	30	31	34	36	42	46	52	60	66	68	77	80	81
CT value								24.4				21.9	26.5		20.2		25.7	
WBC count, ($\times 10^9$/L)	3.4-11.2	10.5	14.12	18.36	15.4	21.22	12.86	21.79	23.53	15.57	15.23	15.72	14.18	9.6	8.82	14.43	11.48	14.04
Neutrophil count, ($\times 10^9$/L)	1.8-7.0	7.6	12.86	15.97	11.99	19.1	12.86	19.02	22.12	14.57	13.07	14.16	12.39	7.5	6.33	12.43	10.23	12.12
Neutrophils (%)	45-75	72.4	90	87	79.6	88	91.5	87.3	94.0	88.0	85.8	84.5	87.4	78.2	71.7	86.2	89.1	86.3
Lymphocyte count, ($\times 10^9$/L)	1.18-3.74	1.4	0.57	1.1	0.81	0.85	0.39	0.67	0.71	0.60	0.55	0.74	0.50	0.99	1.13	0.61	0.46	1.08
Lymphocytes (%)	20-50	13.1	3	6	5.4	4	2.7	3.1	3.0	3.6	3.6	4.5	3.5	10.3	12.8	4.2	4.0	7.7
D- Dimer (ng/mL)	0-229		<150			<150						161		156				
CRP (mg/dL)	<=0.9			8.7	4.9	13.7		14	20	11.3	9.1							
ESR (mm/hr)	0-20		59			49												
LDH (U/L)	118-230	183			289	386												
Procalcitonin (ng/mL)	<=0.08		0.72			0.75		0.49		0.29							0.61	

Abbreviations. CRP: C-reactive protein; CT: cycle threshold; ESR: Erythrocyte sedimentation rate; IL-6: Interleukin-6; LDH: Lactate dehydrogenase; WBC: White blood cells; ** Indicates that no other laboratory values were available on the first day of SARS-CoV2 infection (Day 0).

Supplementary Table 6. Virus isolation from nasopharyngeal swabs of immunocompromised patients.

Patient ID	Viral load (Ct value)	Days post-diagnosis	Virus isolation	Passages in Vero E6 TMPRSS2	Consensus mutations (clinical sample vs prototype strain)	Consensus mutations in nsp5/nsp12 (clinical sample vs passage 3)	Clade
11595	20.7	38	yes	3	nsp12 M794I, Spike E484Q, Spike Q677H	nsp12 Q444K, nsp12 I794M, nsp12 M900V	20G
16902	NA	0	yes	3	No mutations	No mutations	21K
	NA	1	no	-	-	-	-
	NA	7	no	-	-	-	-
	NA	11	no	-	-	-	-
	NA	18	no	-	-	-	-
	19.10	42	no	-	-	-	-
	25.4	50	no	-	-	-	-
17072	26.5	60	no	-	-	-	-
	NA	77	yes	3	nsp5 T169I, nsp12 V223I, nsp12 V792I	No mutations	21K
	25.7	80	no	-	-	-	-
	NA	81	yes	3	nsp5 T169I, nsp12 V223I, nsp12 V792I	nsp5 M165I, nsp5 I169T, nsp12 I792V	21K

Note: NA: Not available; - not available as no virus was isolated.

Supplementary Table 7. The IC_{50} values (μM) of nirmatrelvir and remdesivir against SARS-CoV-2 isolates WT and $nsp5^{T169I}nsp12^{V792I}$ in this study.

	Pre-treatment		Simultaneous treatment		Post-treatment	
Nirmatrelvir	IC_{50} (μM)	Fold	IC_{50} (μM)	Fold	IC_{50} (μM)	Fold
WT	1.77	1	0.95	1	1.67	1
$nsp5^{T169I}nsp12^{V792I}$	3.93	2.22	2.89	3.04	3.62	2.17
Remdesivir	IC_{50} (μM)	Fold	IC_{50} (μM)	Fold	IC_{50} (μM)	Fold
WT	8.8	1	6.68	1	7.5	1
$nsp5^{T169I}nsp12^{V792I}$	16.7	1.9	15.29	2.34	14.56	1.94

Supplementary Table 8. Binding energies (in kcal/mol) of nirmatrelvir drug molecule against nsp5 protein of WT and nsp5^{T169I}nsp12^{V792I} isolates. Hydrogen and hydrophobic interactions are highlighted, providing insights into the molecular interactions at the binding interface.

	Binding Energy (kcal/mol)	H-bond				Hydrophobic Interactions
		Residue No.	No. of Bonds	Molecules	Bond Length (A^0)	
nsp5 of WT docked with nirmatrelvir	-7.8	His163	2	NE2-F3	3.16	Met49, Leu141, Asn142, Ser144, Cys145, His164, Met165, Asp187, Arg188, Gln189, Gln192
				NE2-F2	3.17	
		Glu166	2	N-O2	3.10	
				O-N2	3.25	
nsp5 of nsp5^{T169I}nsp12^{V792I} docked with nirmatrelvir	-6.9	Gly143	1	N-O1	3.01	His41, Met49, Phe140, Leu141, Asn142, His164, Met165, Gln189
		Cys145	1	SG-O1	3.03	
		Glu166	1	N-O3	3.25	

Supplementary Table 9. Binding energies (in kcal/mol) of remdesivir drug molecules against nsp12 protein of WT and nsp5^{T169I}nsp12^{V792I} isolates. Hydrogen and hydrophobic interactions are highlighted, providing insights into the molecular interactions at the binding interface.

<table>
<tr><th rowspan="2"></th><th rowspan="2">Binding Energy (kcal/mol)</th><th colspan="4">H-bond</th><th rowspan="2">Hydrophobic Interactions</th></tr>
<tr><th>Residue No.</th><th>No. of Bonds</th><th>Molecules</th><th>Bond Length (A^0)</th></tr>
<tr><td rowspan="11">nsp12 of WT docked with RTP</td><td rowspan="11">-7.1</td><td>Asp618</td><td>1</td><td>OD1-N5</td><td>3.13</td><td rowspan="11">Lys621, Ser681, Ser682, Thr687, Asp761,</td></tr>
<tr><td>Tyr619</td><td>1</td><td>O-O2</td><td>2.89</td></tr>
<tr><td>Cys622</td><td>1</td><td>N-O2</td><td>3.12</td></tr>
<tr><td rowspan="2">Asp623</td><td rowspan="2">2</td><td>OD1-O13</td><td>2.84</td></tr>
<tr><td>N-O13</td><td>3.03</td></tr>
<tr><td>Thr680</td><td>1</td><td>OG1-O9</td><td>2.86</td></tr>
<tr><td>Asn691</td><td>1</td><td>ND2-O6</td><td>2.81</td></tr>
<tr><td>Ser759</td><td>1</td><td>OG-O6</td><td>2.84</td></tr>
<tr><td rowspan="2">Asp760</td><td rowspan="2">2</td><td>OD2-O12</td><td>3.08</td></tr>
<tr><td>OD1-O12</td><td>3.29</td></tr>
<tr style="display:none"></tr>
<tr><td rowspan="5">nsp12 of nsp5^{T169I}nsp12^{V792I} docked with RTP</td><td rowspan="5">-7.1</td><td rowspan="2">Asn497</td><td rowspan="2">2</td><td>N-O9</td><td>3.05</td><td rowspan="5">Val495, Asn496, Arg569, Gln573, Leu576, Thr686, Thr687, Gly683, Tyr689</td></tr>
<tr><td>O-O9</td><td>3.18</td></tr>
<tr><td>Ser682</td><td>1</td><td>O-O13</td><td>2.80</td></tr>
<tr><td>Asp684</td><td>1</td><td>O-O13</td><td>2.70</td></tr>
<tr><td>Ala685</td><td>1</td><td>O-O2</td><td>3.22</td></tr>
</table>

Supplementary Table 10. Primers used for whole-genome sequencing of SARS-CoV-2 on the Oxford Nanopore technologies GridION platform.

Name	Sequence	Name	Sequence
24 pool 1	TTCTCCTAAGAAGCTATTAAAATCACATGG	23 R pool 2	TTCGCTGATTTTGGGGTCCA
24 R pool 1	GGCTCTTCCATATAGGCAGCT	23 F pool 2	TTTCCTCTGGCTGTTATGGC
24 F pool 1	TGGGTAGTCTTGTAGTGCGT	21 R pool 2 alt_2	TTGCAGCAGGATCCACAAGA
22 R pool 1	GCTGAGCCACATCAAGCCTA	21 R pool 2	AGCCAGCTATAAAACCTAGCCA
22 F pool 1	CCTCAATGAGGTTGCCAAGA	21 F pool 2 alt_2	TTTCAAACACGTGCAGGCTG
20 R pool 1	CTGCACCAAGTGACATAGTGT	19 F pool 2	ACAGATGCGCAAACAGGTTC
20 F pool 1	GGACCTTGAAGGAAAACAGGGT	17 R pool 2	TGTCACTACAAGGCTGTGCA
18 R pool 1	TGGCCATCTTTACACCAAAGC	17 F pool 2 alt omi	TGTGTACATTGGCGACCCTG
18 F pool 1	TGCGGCTTGTAGAAAGGTTCA	15 R pool 2	GCCTCATAAAACTCAGGTTCCC
16 R pool 1	ACAATTTCAGCAGGACAACGC	15 F pool 2	ATGCACGCTGCTTCTGGTAA
16 R pool 1 alt	AGGACAACGCCGACAAGTTC	13 R pool 2	GCAGACGGTACAGACTGTGT
16 F pool 1	TGCTTACCCACTTACTAAACATCCT	13 R pool 2 alt	CCCACAGGGTCATTAGCACA
16 F pool 1 alt	TGAACGGTTCGTGTCTTTAGC	13 F pool 2 alt_2	TCTTGTGCTGCCGGTACTAC
14 R pool 1	GCAGCATTACCATCCTGAGC	11 R pool 2 alt_2	TGGCTGCTGTTGTAAGAGGT
14 F pool 1	ATCCTTTGGTGGTGCATCGT	11,855 F pool 2	GTTGGGTGTTGGTGGCAAAC
12 R pool 1_alt_2	GCAAGTACAAACCTACCTCCCT	11 F pool 2 alt	ATTGTTGGGTGTTGGTGGCA
12 F pool 1 alt_2	AATTTGACCGTGATGCAGCC	9 R pool 2 alt omi	YCTCATAGCACATTGGTAAACAC
10 R pool 1	CTGGACACATTGAGCCCACA	9 F pool 2	TTTTGTCGTGCCTGGTTTGC
10 F pool 1	GACACCTAAGTATAAGTTTGTTCGC	9 F pool 2 alt	GCCCATTGATTGCTGCAGTC
8 R pool 1	GCTGATGTTGCAAAGTCAGTGT	7 F pool 2 alt omi	ACCAACCATATCCAAACGCA
8 F pool 1	GCCCCGATTTCAGCTATGGT	7 R pool 2 alt omi	TGCAAAAGCCTTTACCTCCA
6 R pool 1	TCAATAGCCACCACATCACCA	5 R pool 2 alt_2	GTTCATACTGAGCAGGTGGTG
6 R pool 1 alt	CAATAGCCACCACATCACCA	5 F pool 2 alt_2	ACGTGTTGAGGCTTTTGAGT
6 F pool 1 alt_2	GCTGTTATGTACATGGGCACAC	3 R pool 2	ACCGAGCAGCTTCTTCCAAA
4 R pool 1 alt_2	TGCTGACATGTACCTACCCAG	3 F pool 2	GTGAAGAAGAAGAGTTTGAGCCA
4 F pool 1	GGTGTGGTTGATTATGGTGCT	1 R pool 2	GACCTTCGGAACCTTCTCCA
4 F pool 1 alt	GGGTGTGGTTGATTATGGTGCT	1 F pool 2_alt_omi	ACCAACCAACTTTYGATCTCT
2 R pool 1	GCAGAAGTGGCACCAAATTCC	19 R pool 2 omi	TCTACCAATGGTTCTAAAGCCG
2 F pool 1	TTCTTCGTAAGGGTGGTCGC		

Supplementary Figures:

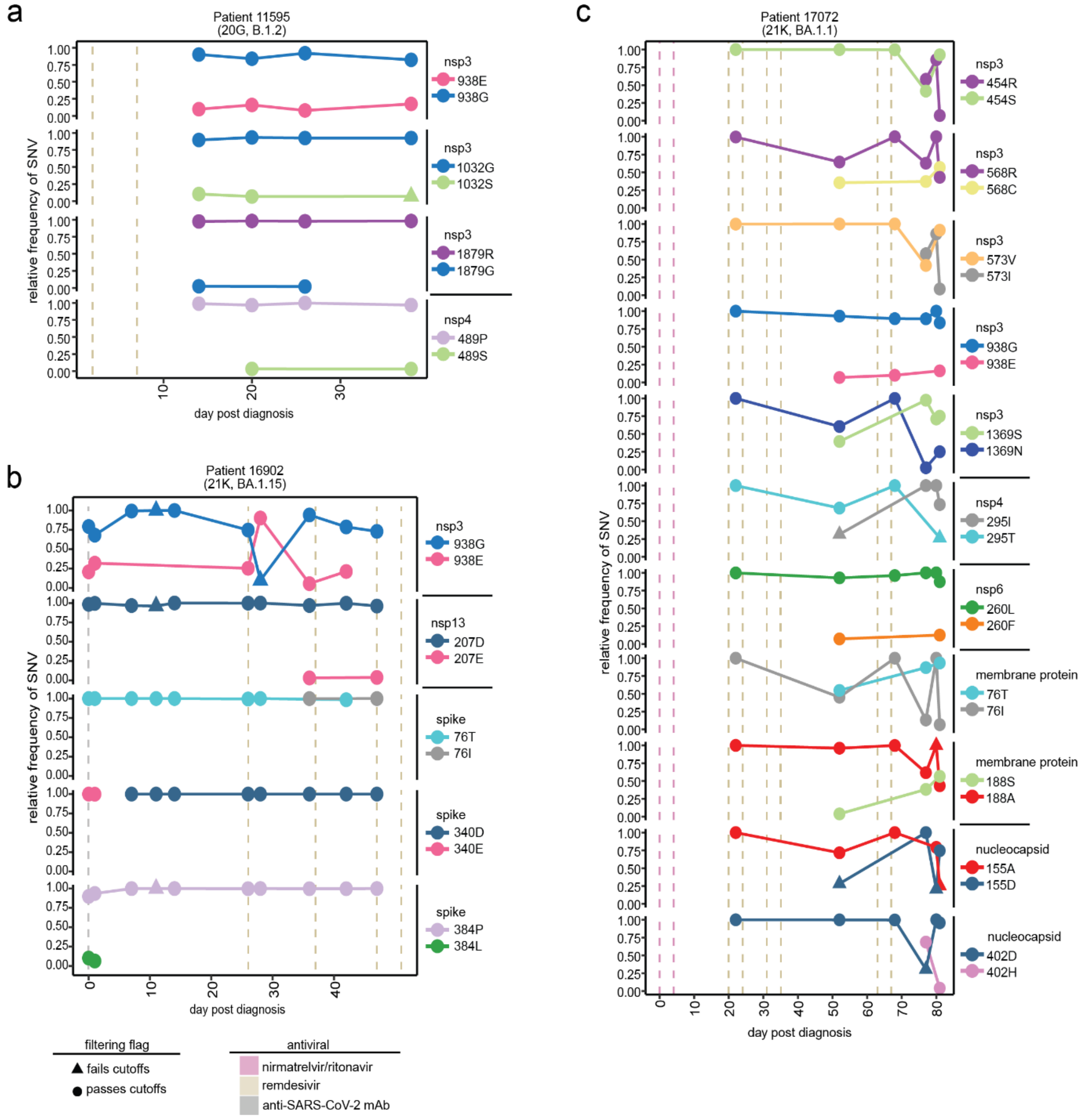

Supplementary Figure 1. **Longitudinal dynamics of non-synonymous SARS-CoV-2 variants.** The relative frequency (y-axis) of non-synonymous SARS-CoV-2 single-nucleotide variants (SNVs) that were present at multiple timepoints throughout the infection for patients (**a**) 11595, (**b**) 16902, and (**c**) 17072. Variant data are grouped by the coding region, amino acid position, and amino acid, with the color of each point and line representing the amino acid and the shape indicating whether the variant was found above (passes, circle) or below (fails, triangle) our detection cutoffs (Methods). All time points along the x-axis are referenced from the date of each patient's initial positive COVID-19 test result, with day 0 marking the date of the first positive test at the New York Presbyterian Hospital. Dashed vertical lines specify each patient's SARS-CoV-2 specific treatment course and are colored based on the treatment category (pink: nirmatrelvir-ritonavir, tan: remdesivir, gray: Sotrovimab). Mutations found in nsp5 and nsp12 are excluded and can be found in Fig. 1b-d. Clade and PANGO lineage designations are included with Patient IDs.

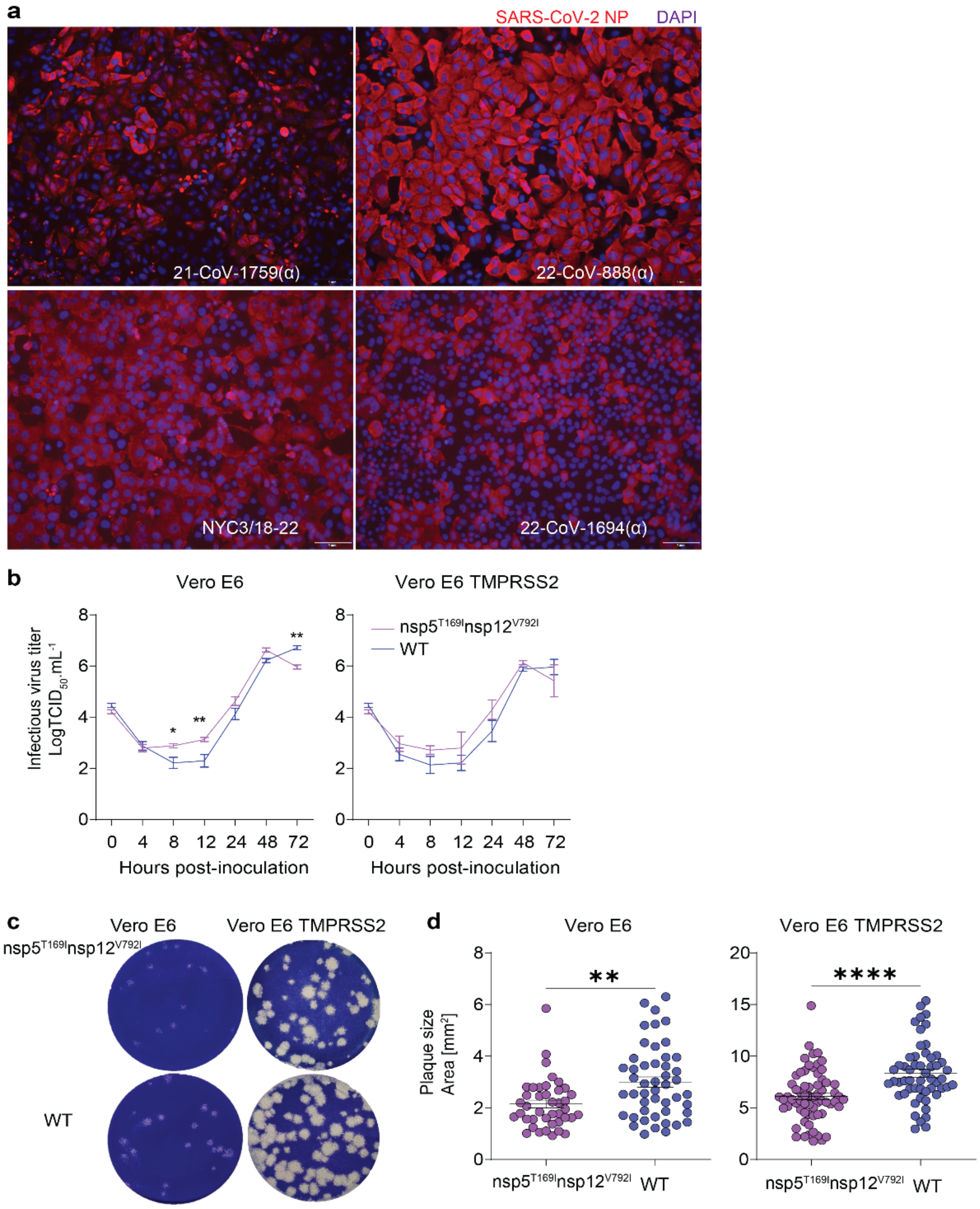

Supplementary Figure 2. **Isolation, growth kinetics and plaque size morphology of SARS-CoV-2 isolates.** (**a**) Immunofluorescence staining confirming the isolation of SARS-CoV-2 isolates from patient samples. Vero E6 TMPRSS2 cells were infected with SARS-CoV-2 isolates (MOI 0.1). After 48 hours, the cell monolayer was fixed and stained with SARS-CoV-2 NP specific monoclonal antibody (red) and counterstained with DAPI (blue). Bar (1 mm) indicates magnification. (**b**) Viral growth kinetics. Vero E6 and Vero E6 TMPRSS2 cells were infected (MOI 0.1) with SARS-CoV-2-nsp5^{T169I}nsp12^{V792I} and WT viruses and virus titers were determined at indicated time points by limiting dilution method and expressed as $TCID_{50}.mL^{-1}$. The data represents mean ± SEM, n = 3, three independent experiments. 2-way ANOVA followed by multiple comparisons test, * $p < 0.05$ and ** $p < 0.01$. (**c**) Viral plaque phenotype. Vero E6 cells were infected (30 plaque forming unit/well) with SARS-CoV-2nsp5^{T169I}nsp12^{V792I} and WT isolates and overlaid with medium containing agar. Plates were incubated at 37 ºC for 72 h, the agar overlay was removed, cells were fixed, and the monolayer was stained with 0.5% crystal violet. Representative images of two independent experiments were shown. (**d**) The diameters of viral plaques from Figure c were measured using a scale in millimeters. The data represent mean ± SEM, n = 41-65, 2 independent experiments. Mann-Whitney U test, ** $p < 0.01$ and **** $p < 0.0001$.

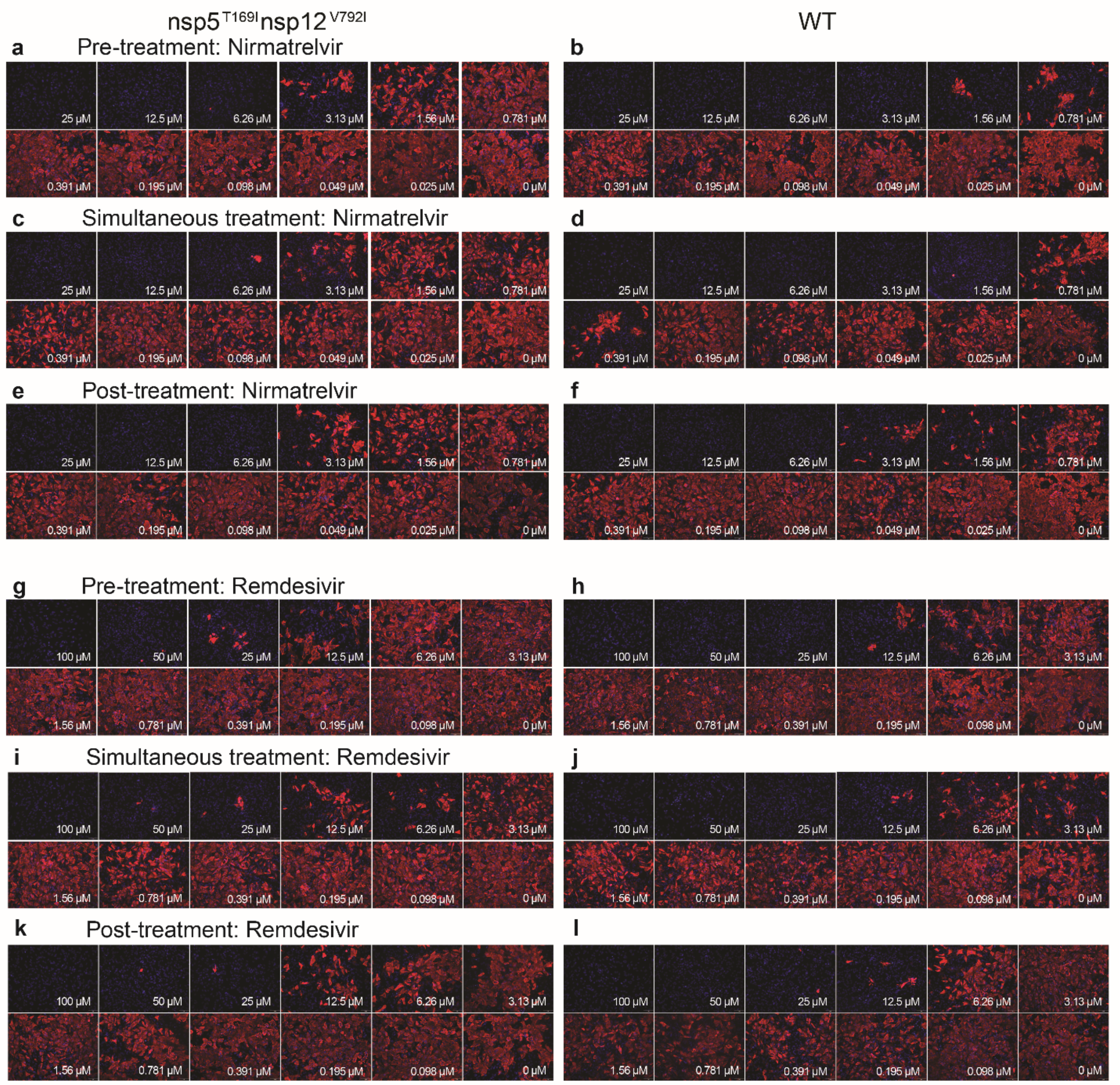

Supplementary Figure 3. **The SARS-CoV-2-nsp5^{T169I}nsp12^{V792I} virus showed decreased sensitivity against nirmatrelvir and remdesivir therapies *in vitro*.** Vero E6 cells were treated with indicated concentration of nirmatrelvir at pre-infection, at infection or at post-infection period and infected with 200 TCID$_{50}$/well of SARS-CoV-2-nsp5^{T169I}nsp12^{V792I} (a, **c, e**) or WT (**b, d, f**). After 48 hours, the cell monolayer was fixed and stained with SARS-CoV-2 NP specific monoclonal antibody (red) and counterstained with DAPI (blue). Vero E6 cells were treated with indicated concentration of remdesivir at pre-treatment, at infection or at post-infection period and infected with 200 TCID$_{50}$/well of SARS-CoV-2nsp5^{T169I}nsp12^{V792I} (**g, i, k**) or WT (**h**, **j**, **l**). After 48 hours, the cell monolayer was fixed and stained with SARS-CoV-2 NP specific monoclonal antibody (red) and counterstained the nucleus with DAPI (blue). (**a-l**) Bar (1 mm) indicates magnification.
