# Supplementary material for: Emergence of transmissible SARS-CoV-2 variants with decreased sensitivity to antivirals in immunocompromised patients with persistent infections
Source: Nat Commun. 2024 Sep 18;15:7999. doi: 10.1038/s41467-024-51924-3 (PMC11411086; doi:10.1038/s41467-024-51924-3)
Supplement: Supplementary file 3 — Reporting Summary [file 41467_2024_51924_MOESM3_ESM.pdf]

nature portfolio

Corresponding author(s): Ghedin, Diel, Salvatore

Last updated by author(s): 8/11/2024

Reporting Summary

Nature Portfolio wishes to improve the reproducibility of the work that we publish. This form provides structure for consistency and transparency in reporting. For further information on Nature Portfolio policies, see our Editorial Policies and the Editorial Policy Checklist.

Statistics

For all statistical analyses, confirm that the following items are present in the figure legend, table legend, main text, or Methods section.

n/a	Confirmed	
☐	☒	The exact sample size (*n*) for each experimental group/condition, given as a discrete number and unit of measurement
☐	☒	A statement on whether measurements were taken from distinct samples or whether the same sample was measured repeatedly
☐	☒	The statistical test(s) used AND whether they are one- or two-sided<br>*Only common tests should be described solely by name; describe more complex techniques in the Methods section.*
☒	☐	A description of all covariates tested
☒	☐	A description of any assumptions or corrections, such as tests of normality and adjustment for multiple comparisons
☐	☒	A full description of the statistical parameters including central tendency (e.g. means) or other basic estimates (e.g. regression coefficient) AND variation (e.g. standard deviation) or associated estimates of uncertainty (e.g. confidence intervals)
☒	☐	For null hypothesis testing, the test statistic (e.g. *F*, *t*, *r*) with confidence intervals, effect sizes, degrees of freedom and *P* value noted<br>*Give P values as exact values whenever suitable.*
☒	☐	For Bayesian analysis, information on the choice of priors and Markov chain Monte Carlo settings
☒	☐	For hierarchical and complex designs, identification of the appropriate level for tests and full reporting of outcomes
☒	☐	Estimates of effect sizes (e.g. Cohen's *d*, Pearson's *r*), indicating how they were calculated

Our web collection on statistics for biologists contains articles on many of the points above.

Software and code

Policy information about availability of computer code

Data collection	No software was used.
Data analysis	online SWISS model server; PyMOL Molecular Graphics System, Version 2.3.4, Schrodinger, LLC; LIGPLOT+; GraphPad Prism 9 (GraphPad, La Jolla, CA, USA), bwa-mem (v.0.7.17), fgbio (v.2.0.2), snakemake (v.6.12.3), timo (v4, https://github.com/GhedinSGS/timo), trimmomatic (v.0.39), iVar (v.1.3.1), GATK, NextClade, R (4.2.3). Analysis code is available at https://doi.org/10.5281/zenodo.13306528 (v1) and https://github.com/GhedinSGS/SARS-CoV-2_Antiviral_Resistance.

For manuscripts utilizing custom algorithms or software that are central to the research but not yet described in published literature, software must be made available to editors and reviewers. We strongly encourage code deposition in a community repository (e.g. GitHub). See the Nature Portfolio guidelines for submitting code & software for further information.

Data

Policy information about availability of data

All manuscripts must include a data availability statement. This statement should provide the following information, where applicable:

- Accession codes, unique identifiers, or web links for publicly available datasets
- A description of any restrictions on data availability
- For clinical datasets or third party data, please ensure that the statement adheres to our policy

Additional clinical data of the patients presented in this article are not readily available because of limitations protecting proprietary information. Requests to access additional data should be directed to :mis2053@med.cornell.edu

Research involving human participants, their data, or biological material

Policy information about studies with human participants or human data. See also policy information about sex, gender (identity/presentation), and sexual orientation and race, ethnicity and racism.

Reporting on sex and gender	Sex of study participants is reported in the manuscript
Reporting on race, ethnicity, or other socially relevant groupings	Race of study participants is not available/not reported
Population characteristics	N/A
Recruitment	Patients were either recruited directly because immunocompromised and affected by COVID-19 and/or samples were obtained from Weill Cornell Biobank
Ethics oversight	Weill Cornell Medicine IRB

Note that full information on the approval of the study protocol must also be provided in the manuscript.

Field-specific reporting

Please select the one below that is the best fit for your research. If you are not sure, read the appropriate sections before making your selection.

☒ Life sciences ☐ Behavioural & social sciences ☐ Ecological, evolutionary & environmental sciences

For a reference copy of the document with all sections, see nature.com/documents/nr-reporting-summary-flat.pdf

Life sciences study design

All studies must disclose on these points even when the disclosure is negative.

Sample size	For transmission study in hamsters, four animals per group were used. Nasopharyngeal swabs were collected longitudinally from fifteen immunocompromised patients, for a total of 67 samples.
Data exclusions	Data with poor SARS-CoV-2 amplification (<75% of the genome covered at 5X) were excluded from variant analyses.
Replication	The in vitro antiviral resistance analysis studies included two technical replicates and 2-3 independent experiments. Illumina libraries were generated in duplicate using the same aliquot of viral RNA.
Randomization	Animals were randomly allocated into four different groups keeping equal numbers of male and female.
Blinding	Blinding during animal study was not possible as working with BSL3 agents requires identification of individual cages with the infectious agents, doses, routes, animal IDs, etc.

Reporting for specific materials, systems and methods

We require information from authors about some types of materials, experimental systems and methods used in many studies. Here, indicate whether each material, system or method listed is relevant to your study. If you are not sure if a list item applies to your research, read the appropriate section before selecting a response.

Materials & experimental systems

n/a	Involved in the study	
☐	☒	Antibodies
☐	☒	Eukaryotic cell lines
☒	☐	Palaeontology and archaeology
☐	☒	Animals and other organisms
☐	☒	Clinical data
☒	☐	Dual use research of concern
☒	☐	Plants

Methods

n/a	Involved in the study	
☒	☐	ChIP-seq
☒	☐	Flow cytometry
☒	☐	MRI-based neuroimaging

Antibodies

Antibodies used	SARS-CoV-2 anti-N mAb, mouse, clone B61G11, Custom made
Validation	The antibody was used in several recent publications (10.1128/jvi.01902-23, 10.1073/pnas.2215067120, 10.1128/jvi.00961-22)

Eukaryotic cell lines

Policy information about cell lines and Sex and Gender in Research

Cell line source(s)	Vero E6 (ATCC® CRL-1586™) and Vero E6 TMPRSS2 (JCRB Cell Bank, JCRB1819)
Authentication	Cells were obtained from commercial sources.
Mycoplasma contamination	Cells were periodically treated with antimycoplasmal drugs. No mycoplasmal test was performed.
Commonly misidentified lines (See ICLAC register)	N/A

Animals and other research organisms

Policy information about studies involving animals; ARRIVE guidelines recommended for reporting animal research, and Sex and Gender in Research

Laboratory animals	LVG golden Syrian hamsters, strain 049, 7 weeks old
Wild animals	No wild animals were used.
Reporting on sex	Equal number of both male and female was used in the animal experiment.
Field-collected samples	The study did not involve samples collected from field animals.
Ethics oversight	The study procedures were reviewed and approved by the Institutional Animal Care and Use Committee at Cornell University (IACUC approval number 2021-0021).

Note that full information on the approval of the study protocol must also be provided in the manuscript.

Clinical data

Policy information about clinical studies

All manuscripts should comply with the ICMJEguidelines for publication of clinical research and a completedCONSORT checklist must be included with all submissions.

Clinical trial registration	N/A
Study protocol	N/A
Data collection	N/A
Outcomes	N/A

Plants

Seed stocks	N/A
Novel plant genotypes	N/A
Authentication	N/A
